# Cytokine Profile in Patients with Aseptic Loosening of Total Hip Replacements and Its Relation to Metal Release and Metal Allergy

**DOI:** 10.3390/jcm8081259

**Published:** 2019-08-20

**Authors:** Rune J. Christiansen, Henrik J. Münch, Charlotte M. Bonefeld, Jacob P. Thyssen, Jens J. Sloth, Carsten Geisler, Kjeld Søballe, Morten S. Jellesen, Stig S. Jakobsen

**Affiliations:** 1 Department of Mechanical Engineering, Technical University of Denmark, DK-2800 Kgs. Lyngby, Denmark; 2 Department of Immunology and Microbiology, University of Copenhagen, DK-2200 Copenhagen, Denmark; 3 Institute of Clinical Medicine—Orthopedic Surgery, Aarhus University, DK-8000 Aarhus C, Denmark; 4 Institute of Clinical Medicine, Copenhagen University, Gentofte Hospital, DK-2900 Hellerup, Denmark; 5 National Food Institute, Research Group on Nanobio Science, Technical University of Denmark, DK-2860 Søborg, Denmark

**Keywords:** arthroplasty, replacement, hip, hypersensitivity, contact, allergy and immunology, cytokines, Interleukin-8

## Abstract

Metal release from total hip replacements (THRs) is associated with aseptic loosening (AL). It has been proposed that the underlying immunological response is caused by a delayed type IV hypersensitivity-like reaction to metals, i.e., metal allergy. The purpose of this study was to investigate the immunological response in patients with AL in relation to metal release and the prevalence of metal allergy. THR patients undergoing revision surgery due to AL or mechanical implant failures were included in the study along with a control group consisting of primary THR patients. Comprehensive cytokine analyses were performed on serum and periimplant tissue samples along with metal analysis using inductive coupled plasma mass spectrometry (ICP-MS). Patient patch testing was done with a series of metals related to orthopedic implant. A distinct cytokine profile was found in the periimplant tissue of patients with AL. Significantly increased levels of the proinflammatory cytokines IL-1β, IL-2, IL-8, IFN-γ and TNF-α, but also the anti-inflammatory IL-10 were detected. A general increase of metal concentrations in the periimplant tissue was observed in both revision groups, while Cr was significantly increased in patient serum with AL. No difference in the prevalence of metal sensitivity was established by patch testing. Increased levels of IL-1β, IL-8, and TNF-α point to an innate immune response. However, the presence of IL-2 and IFN-γ indicates additional involvement of T cell-mediated response in patients with AL, although this could not be detected by patch testing.

## 1. Introduction

### 1.1. Background

Aseptic loosening (AL) of implants is the most common reason for revision surgeries in patients with total hip replacements (THRs), representing close to 75% of all cases, with serious consequences for patients and healthcare systems [1,2]. Although the etiology of AL is multifactorial and yet to be fully understood, evidence suggest that the predominant cause of AL is due to a macrophage-driven chronic inflammatory response initiated by implant wear [1,3,4,5]. This adverse tissue reaction is associated with the innate immune system and can lead to the bone degrading state of osteolysis, subsequently resulting in implant failure by AL. 

Cytokines are small messenger molecules that coordinate the immune response by regulating inflammation and modulating cellular activities such as growth, differentiation and survival [6]. Key mediators of osteolysis have been identified as pro-inflammatory cytokines like interleukin (IL)-1β, IL-6, IL-8 and tumor necrosis factor (TNF)-α secreted by activated macrophages. In turn, these cytokines are capable of inducing the differentiation of osteoclast precursor cells into mature, bone resorbing, osteoclasts [1,7,8,9,10,11]. Interferon (IFN)-γ is another important immune regulatory cytokine implicated in bone resorption but also in inflammation progression and cell mediated immunity [12,13].

Macrophages have been established as important mediators of ostolysis, but several other cell types have also been identified in the periimplant tissue of failed implants, including lymphocytic T cells [9,14,15,16]. T helper (Th) cells, a subtype of T cells, are important regulators of macrophage function and the adaptive immune response, which given rise to the concept of implant-related metal sensitivity. This concept is evolved around a delayed type IV T cell mediated hypersensitivity (DTH), exemplified by allergic contact dermatitis to metal ions (metal allergy). Due to their small size, metal ions are considered to be incomplete antigens, referred to as haptens, and must interact with peptides or proteins to form an antigen able to mount DTH. 

In support of the concept above, findings of elevated levels of metal particles and ions have been shown to correlate with an increased prevalence of metal allergy in patients with failing implants [17,18,19,20,21]. Furthermore, some of the most commonly applied alloys for THR like stainless steel (FeCrNiMo), cobalt chromium (CoCrMo) and titanium alloys (Ti6Al4V) contain known sensitizing metals [22]. 

Immunological studies of AL in THRs, have suggested the involvement of a Th1 cell response, crucial for DTH, due to increased levels of Th1 cell specific cytokines like IFN-γ and IL-2 [4,23]. Previous studies also suggest the involvement of a Th2 and Th17 cell response in AL, which are respectively characterized by the production of IL-4, IL-17 and granulocyte-macrophage colony-stimulating factor (GM-CSF) [24,25,26]. However, the causal relationship between immune reactions, metal release from implants, and AL is still uncertain. 

### 1.2. Aim

The aim of the present study was to determine and compare levels of THR relevant metals and cytokine profiles from periimplant tissue and blood serum, and to investigate the prevalence of metal allergy in patients undergoing revision surgery due AL, mechanical failure or undergoing primary THR surgery. Periimplant tissue obtained from patients with AL showed a significantly different cytokine profile suggesting the involvement of both innate and adaptive immunity in AL. No prevalence of metal allergy was established in patients with failed implants despite elevated levels of metal ions in the periimplant tissue.

## 2. Experimental Section

### 2.1. Patients and Samples

We conducted a prospective case study including three patient groups. This study was approved by the Central Denmark Region Committee on Biomedical Research Ethics (Journal number: 1-10-72-90-13). All patients gave their written informed consent before entering the study. 

Criteria for inclusion in the AL (+) group were: revision (entirely or partial) due to aseptic loosening, osteolysis, or unexplainable pain that could not be treated conservatively. The AL (−) group; revision (entirely or partial) due to fracture, dislocation, or component failure. The Control group; patients received a primary THR. Implant components are listed in Table 1. 

Criteria for exclusion: infection (positive Kamme-Lindberg biopsies [27]), use of immunomodulating medication, occupational metal exposure, known metal allergies towards implanted metals or secondary osteoarthritis (fracture, inflammation). The mean age for the AL (+) group was 60.8 years with a gender distribution of 4/2 (M/W). For the AL (−) group, the mean age was 73 years, and the distribution was 4/2 (M/W). The control group had a mean age of 62 years and a distribution of 5/3 (M/W). Tissue samples for cytokine and ICP-MS analysis were snap-frozen in liquid nitrogen and stored at −80 °C for later use. Serum obtained from patients blood samples were taken before the operation and stored at −80 °C for later cytokine and ICP-MS analysis.

### 2.2. Cytokine Profile Analysis

Snap frozen tissue samples from group AL (+), group AL (−) and the control group were mechanically disrupted and homogenized (Precellys®24 and Cryolys®—Bertin Technologies, Bie & Berntsen A/S 2730, Herlev, Denmark) at 4 °C for 4 × 20 s in lysis buffer containing protease inhibitor cocktail (REF 11836145001, Roche Diagnostics, Indianapolis, IN, USA). Homogenized tissue samples were then spun for 10 min at 10,000 × G at 4 °C (Microcentrifuge 157MP—Ole Dich Instrumentmakers ApS, Hvidovre, Denmark) and the protein concentration in the supernatant was estimated by Bradford protein assay [28] using Coomassie blue (#1610436. Bio-Rad Laboratories, Inc., Hercules, CA, USA). Prior to cytokine analysis, total protein concentrations of the samples were adjusted to 0.5 mg/mL. Cytokine analysis was performed using a validated V-PLEX electrochemiluminescence immunoassays (Meso Scale Discovery, Rockville, MD, USA). A total of 11 cytokines divided on two separate kits were analyzed. Proinflammatory Panel 1 contained; IL-1β, IL-2, IL-4, IL-6, IL-8, IL-10, IFN-γ and TNF-α (catalog # K15049D-1), and cytokine panel 1 kit contained; IL-15, IL-17A, GM-CSF (catalog # K15050D-1). Samples were analyzed in triplicates (MESO QuickPlex SQ 120—Meso Scale Discovery). Calibration curves used to calculate cytokine concentrations were established by fitting to a 4 parameters logistic model with a 1/Y^2^ weighting. Cytokine concentrations were calculated using the Discovery workbench 4.0.12 software (Meso Scale Discovery). Serum samples were analyzed undiluted using the same cytokine kits as used for the tissue.

### 2.3. Patch Testing

A special patch test series, provided by Smart Practice® (Phoenix, AZ, USA), was used in this study. The patch contained prefabricated panels with metallic compounds associated with orthopedic prostheses on Scanpor tape. Standard metal allergens included; nickel (II) sulphate NiSO_4_ (1.0 wt.%), potassium dichromate (VI) K_2_Cr_2_O_7_ (0.054 wt.%) and cobalt (II) chloride CoCl_2_ (0.02 wt.%). In addition, a customized panel with the following metals and corresponding titrations were included; vanadium (IV) oxide sulfate hydrate VOSO_4_·H_2_O (0.36, 0.18, 0.06, 0.02 wt.%), vanadium (III) chloride VCl_3_ (0.24, 0.12, 0.013, 0.04 wt.%), manganese (II) chloride MnCl_2·_4H_2_O (0.24, 0.08, 0.06, 0.0057 wt.%), aluminum (III) chloride AlCl_3_·6H_2_O (0.72, 0.38, 0.039 wt.%), ammonium molybdate (VI) (NH_4_)_6_Mo_7_O_24_ 4H_2_O (0.12, 0.013, 0.04 wt.%), titanium (IV) oxalate hydrate TiC_4_O_8_·H_2_O (0.32, 0.16, 0.08, 0.04 wt.%), titanium (IV) dioxide TiO_2_ (0.24 wt.%), potassium titanium (II) oxide oxalate C_4_K_2_O_9_Ti·2H_2_O (2.4, 1.2, 0.6 wt.%), ammonium titanium (II) lactate, solution Ti [(C_3_H_4_O_3_)_2_(NH_4_OH)_2_] (0.16, 0.08, 0.04 wt.%), ammonium titanium (IV) peroxocitrate (NH_4_)_4_[Ti_2_(C_6_H_4_O_7_)_2_(O_2_)_2_]·4H_2_O (0.32, 0.16, 0.08, 0.04 wt.%). methyl methacrylate C_5_H_8_O_2_ (2 wt.%), gentamycin sulfate (20 wt.%) and ferrous chloride FeCl_2_ (2 wt.%) were tested by manually loading of a Finn chamber on Scanpor tape. Patches were applied on the upper back and were occluded for 48 h. Readings were completed 96 h after application [29]. The patients were instructed to remove the panels after 48 h, and not to shower, scratch or expose to sunlight. Reactions were scored using the International Contact Dermatitis Research Group’s (ICDRG) criteria [30]. Only definite +1, +2 and +3 reactions were regarded as positive.

### 2.4. ICP-MS (Serum)

Blood samples were sent to Vejle Hospital, Department of Clinical Biochemistry, Denmark, for determination of chromium and cobalt levels before the surgery. The samples were analyzed by ICP-MS instrument (iCAPq, Thermo Fisher Scientific Inc., Waltham, MA, USA). The samples were diluted with 0.5% HNO_3_, gallium was added as an internal standard prior to analysis. The detection limit was 10 nmol/L equivalent to 0.59 ppb (cobalt) and 0.52 ppb (chromium).

### 2.5. ICP-MS (Tissue and Serum)

Elemental analysis of tissues and titanium (Ti) analysis of blood was performed at the National Food Institute at the Technical University of Denmark.

*Elemental analysis in tissues:* Tissue samples (0.1–0.5 g) were digested with a mixture of concentrated nitric acid (4 mL; PlasmaPure, SCPScience, Courtaboeuf, France) and hydrogenperoxide (1 mL; Merck, Darmstadt, Germany) in a microwave oven (Multiwave 3000, Anton Paar, Graz, Austria). The concentration of aluminum (Al), vanadium (V), chromium (Cr), cobalt (Co) and nickel (Ni) was determined using ICPMS (iCAPq, Thermo Fisher Scientific, Waltham, MA, USA) using rhodium as an internal standard and external calibration. The ICPMS instrument was run in the kinetic energy discrimination (KED) mode using helium as a collision cell gas. The limit of detection was estimated at 100 µg/kg for all elements.

*Determination of Ti in tissue and blood:* The acid digests of tissues were also subjected to Ti analysis. Serum subsamples (200 µL) were diluted with 4.8 mL diluent solution consisting of 0.5% Triton X-100, 10% ethanol (both Merck) and 1% nitric acid (SCPScience) prior to the analysis of the concentration of Ti using a triple quadrupole ICPMS (Agilent 8800 ICP-QQQ, Agilent Technologies, Yokogawa, Japan) and using ammonia as a cell gas with determination of Ti after MS/MS mass shift from *m/z* 48 ≥ *m/z* 150 with scandium (Sc) as internal standard and external calibration. The data quality of Ti analysis was assessed by the analysis of the reference material Seronorm (Sero, Oslo, Norway). The obtained value 7.2 µg/L was in good agreement with the reference value 6.8 µg/L. The limit of detection was estimated at 1 µg/L in serum samples and 20 µg/kg in tissues. All calibration standards and internal standards were produced from certified single-element stock solutions (SCPScience).

### 2.6. Statistical Analysis 

For group comparison the Kruskal-Wallis test was used, and if statistically significant, the Mann-Whitney U test was used to compare between individual groups. By convention, to calculate group medians, metal concentrations below the detection limit were assigned a value of one-half the detection limit. Comparisons were made using the Mann-Whitney test. Contingency tables (patch test) were analyzed using Fisher’s exact test. A significance level of *p* < 0.05 was considered statistically significant. Matlab R2014a (8.3.0.532) with statistical toolbox (MathWorks Inc. Natick, MA, USA) was used for statistical analysis. For graphical representation Prism 6.0 (GraphPad Software, San Diego, CA, USA) was used.

## 3. Results

### 3.1. Cytokine Profile Analysis 

#### 3.1.1. Analysis of Cytokine Levels in Periimplant Tissue

**Figure 1:**
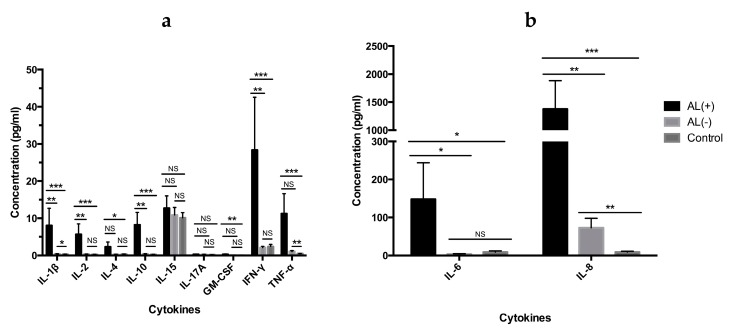
Cytokine profiles of periimplant tissue. Cytokines are shown in graph (**a**) and (**b**) with different concentration scales. Except from IL-15 and IL-17, patients with aseptic loosening AL (+) showed a statistically significant increase in the cytokine levels when compared with the control group. Out of the statistically significant cytokines, IL-4 and TNF-α did not show any statistical significance (NS) when comparing the two revision groups. IL-8 was found to be highly increased in patients with AL. Results are expressed as the mean (±SEM). The Mann-Whitney U test was used for the statistical analysis with a significance level of 0.05. *p* values are given by * *p* < 0.05, ** *p* ≤ 0.01, *** *p* ≤ 0.001.

Cytokine levels were measured in periimplant tissue obtained from revision or primary surgery to identify a potential local immune response (Figure 1). 

Altogether, 10 cytokines (IL-1β, IL-2, IL-4, IL-6, IL-8, IL-10, IL-15, IL-17A, IFN-γ and TNF-α) and growth factor GM-CSF were analyzed (Figure 1a/b). We found a highly increased cytokine profile in patients with AL, with a statistical significant increase of IL-1β, IL-2, IL-4, IL-6, IL-8, IL-10, GM-CSF, IFN-γ and TNF-α when compared to the AL (+) and the control group. When compared to the AL (−) group we found a statistically significant increase for all cytokines except from IL-4, IL-15, GM-CSF, and TNF-α. Of note, IL-8 was highly increased and the most strongly associated cytokine with AL.

#### 3.1.2. Analysis of Cytokine Levels in Serum

**Figure 2:**
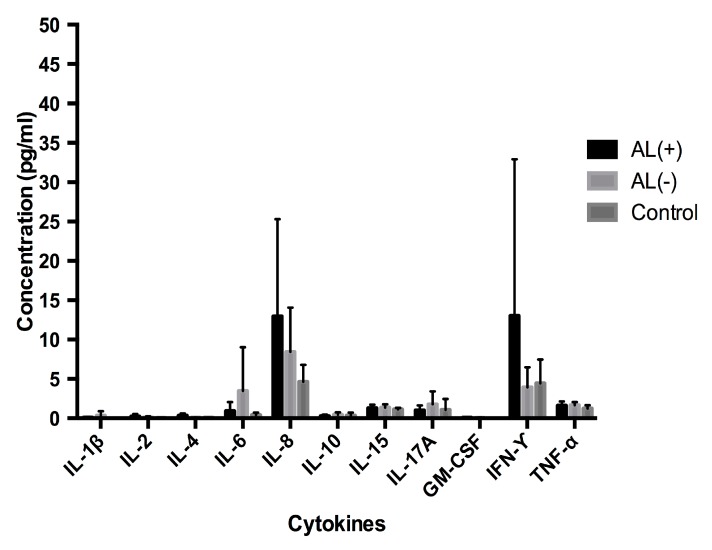
Cytokine profiles in serum. Patients with aseptic loosening are represented as AL (+), patients with dislocations are represented as AL (−) and the controls. Increased IL-8 and IFN-ϒ levels appeared for the AL (+) group. Results are expressed as the mean concentration (±SEM). No statistically significant differences could be established between the groups using the Mann-Whitney U test with a significance level of 0.05.

An identical cytokine profile analysis was performed in serum to investigate a corresponding systemic response (Figure 2). Cytokine levels in serum appeared 10–100 fold lower and although IL-8 and IFN-γ seemed increased in the AL (+) group, no statistical differences could be established. Together these results show a general increase of the investigated cytokine profile, in periimplant tissue obtained from patients with AL, but also that cytokine levels in periimplant tissue are not necessarily reflected in blood serum. Among other increased cytokines, IL-8 was established as the most potent marker of AL.

### 3.2. Patch Test

**Figure 3:**
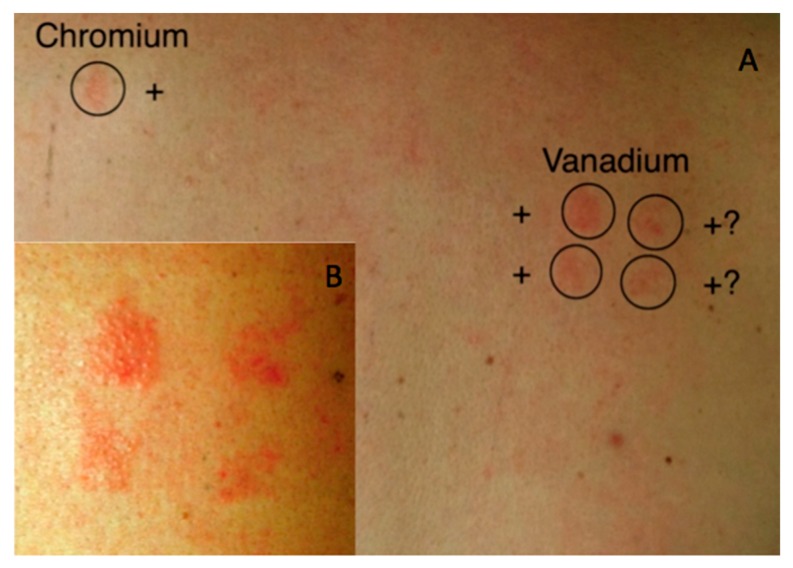
Patch test. (**A**) Example of a positive (+) and a doubtful skin reactions (+?) to vanadium and chromium in a patient from the AL (+) group. (**B**) Enlarged photograph of the skin reaction to vanadium.

All patient groups were subjected to a comprehensive patch test containing orthopedically relevant metals and methyl methacrylate, the monomer of poly (methyl methacrylate) (PMMA) used as bone cement in THR (Table 2). Positive and doubtful reactions to these metals are summarized in Table 2. No statistical significant differences between either of the groups could be established. Few positive test reaction were observed even for the metals used in the standard series (Cr, Co and Ni), only one reaction to Ni and one to Cr were observed in all three groups. However, three positive reactions for Ti and two positive skin reactions to V were observed in the (AL+) group. In fact, the two positive reactions to V were observed in the same patient who had a positive reaction to Cr (Figure 3). 

### 3.3. ICP-MS Analysis

#### 3.3.1. Metal Concentrations in Periimplant Tissue

Periimplant tissue was analyzed for Al, Ti, V, Cr, Co and Ni by ICP-MS (Table 3). Raised median concentrations of most metals could be observed in both revision groups, AL (+) and AL (−) as shown in Table 3. Metals found at highest concentrations were, Al, Ti and Cr, although no statistically significant differences could be established between the AL (+) and AL (−) group, however, a difference was observed when compared to the control group. Despite the raised concentrations of Cr observed in the AL (+) group compared to the control group no statistical significant increase could be determined (*p* = 0.074). These results clearly demonstrate the presence of metal release in the two revision groups. 

#### 3.3.2. Metal Concentrations in Serum

Serum samples were analyzed for Ti, Co and Cr by ICP-MS (Table 3). A statistical significant increase of Cr concentrations in the AL (+) group was found, compared to the control group. No statistical significant increase was observed between the two revision groups (*p* = 0.105). Nevertheless, the highest concentrations of both Co and Cr was found in the AL (+) group. One patient in the control group showed a high concentration of Ti and despite reanalysis, this sample still showed a high Ti concentration, preventing it from being regarded as an outlier. All other Ti concentrations in the control group were at the detection limit of the ICP-MS method. Furthermore, the results show that local metal concentrations in the periimplant tissue can be highly increased compared to serum levels.

## 4. Discussion

The possibility of metal allergy leading to aseptic loosening has been debated in the literature for many years [21,31,32,33]. Still, the long-term effect of internally released metals remains unknown and so does the underlining immunological response lead to AL and implant failure [22]. In this study we investigated the correlations between the immunological profile, metal allergy and metal released from implants, in THR patients with AL. 

We found that patients with AL had a cytokine profile with statistically significant increased levels of the pro-inflammatory cytokines IL-1β, IL-6, and IL-8, but also Th1 associated cytokines, IL-2 and IFN-γ, and the anti-inflammatory cytokine IL-10, when compared to patients with implant failures due to mechanical causes. Despite a statistically significant and substantial metal exposure both locally and systemically in THR patients, we were not able to prove any systemic effect by cytokine analysis of serum or by positive patch testing. Based on the present study, a systemic effect cannot be ruled out due to the low number of patients enrolled in this study. The findings are, however, in line with the clinical observations, where the adverse effect to implants is predominantly observed locally rather than systemically. A further limitation of this study was the clinical approach, where polyethylene (PE) debris derived from the acetabular liner is most likely contributing the innate part of the cytokine profile observed in the periimplant tissue.

Cytokines play an important role in AL, not only as regulators of osteolysis, but also as important identifiers of the occurring immune response. In our cytokine analysis we included IL-1β IL-2, IL-4, IL-6, IL-8, IL-10, IL-15, IL-17A, GM-CSF, IFN-γ and TNF-α due to their implication in innate and adaptive immunity and their function as osteolytic mediators (Figure 1 and Figure 2). In addition to being involved in the innate immune response, IL-1β, IL-6, IL-8, GM-CSF, and TNF-α have previously been identified as mediators of osteolysis [14,34]. In accordance with these observations, we found elevated levels of these cytokines in the periimplant tissue from the AL (+) group when compared to the control group. When comparing the two revision groups, AL (+) and AL (−), no statistically significant difference was seen for GM-CSF and TNF-α. However, levels of GM-CSF were very low and might be considered without any biological effect. TNF-α is well-known as a strong inducer of osteolysis and is the first proinflammatory cytokine produced in response to many wear particles and stimulates macrophage production of IL-1β and IL-6 [35]. Although no statistically significance is seen for TNF-α between the two revision groups, both IL-1β and IL-6 still showed a statistically significant increase in the AL (+) group. In comparison, other investigators have found low levels of IL-1β and TNF-α in periimplant tissue from patients with failed THRs due to osteolysis [36]. Moreover, they found that IL-6 and IL-8 were consistent with failed implants, suggesting that IL-6 and IL-8 might be the primary drivers of end-stage osteolysis, while IL-1β and TNF-α are critical mediators in the acute phase of inflammation. Interestingly, these observations did indeed correspond well to our findings of IL-6 and notably IL-8, which we found to be the strongest predictor of AL.

The main IL-8 secreting cells are macrophages, osteoblasts and osteoclasts. Studies have shown that IL-8 holds multiple functions in AL and has been found to affect both neutrophils, T cells, monocyte/macrophages and osteoclasts [37,38]. It has been demonstrated that wear particle stimulation of osteoblasts and macrophages promotes IL-8 production, which in turn can lead to both macrophage activation and induce phagocytosis [39]. Interleukin-8 also possess chemotactic properties on neutrophils and T cells and could conceivably play a role in attracting such cells to the periimplant tissue [37,40]. Moreover, IL-8 is shown to promote osteoclastogenesis and the formation of osteoclasts that are capable of secreting IL-8 on their own. Thus, the high levels of IL-8 observed in patients with AL is probably not only caused by an innate immune response but also in part by the osteolytic process taking place in the patients with AL, which could explain the differences in IL-8 observed between the AL (+) and the AL (−) group [40]. 

As indicators of DTH, IL-2 and IFN-γ levels were statistical significantly increased in the AL (+) group compared to the AL (−), supporting the involvement of a Th1 cell response in AL. This is consistent with other studies, showing lymphocyte reactivity to implant related metals and production of Th1-specific cytokines (IFN-γ and IL-2), and even the generation of metal specific T cells [41,42]. Macrophages are capable of producing IFN-γ but abundant evidence suggests that T cells and natural killer (NK) cells are the major sources of IFN-γ [3,43,44]. Accompanied by the increased levels of IL-2, the increased IFN-γ levels found in patients with AL further support the involvement of a Th1 cell response. Interferon-gamma possess both pro- and anti-inflammatory activities with the functional outcome being dependent on secretion levels, pathogenesis and disease severity [13,44]. Some studies show a protective effect of IFN-γ on osteolysis, possible by inhibiting the early differentiation of osteoclasts, whereas others have shown that IFN-γ promotes osteoclast formation [13]. How IFN-γ affects the progression of AL in this study is difficult to decipher but low levels of IFN-γ does not exert the inhibitory effect on osteoclasts and seems to be limited to the early stage of osteoclast differentiation. Furthermore, IFN-γ can promote osteoclast maturation in the late state of osteoclast formation leading to a shift from the inhibitory effect towards a state of bone resorption [45]. 

In addition to the Th1 signature cytokines, we also observed an increase of IL-4, along with a statistically significant increase of IL-10 when comparing the two revision groups. 

Although the production of these cytokines are related to Th2 cells, IL-10 is also produced by monocytes and regulatory T cells, acting as an anti-inflammatory cytokine, which could regulate cell-mediated reactions involved in AL [46,47,48]. We were not able to detect any consistent cytokine profile at a systemic level in serum, underlining the difficulty of detecting AL based on the systemic levels of cytokines. In fact, cytokines have a short half-life in serum due to their potent nature as signaling molecules, which makes cytokines very challenging to use as biomarkers in serum [49]. 

In our analysis of Ti, Co and Cr in serum, we found a statistically significant increase of Cr in the AL (+) group and Ti in the Al (−) group (Table 3). Furthermore, we did detect a correlation between raised Ti concentrations in serum from patients with a stem component made from a Ti containing alloy, which corresponds to the findings of other studies applying the ICP-MS method [50]. Metal release, has previously been shown to increase in patients with poorly functioning implants [17]. From a corrosion point of view, this could be explained by increased micro-motions of the implant leading to fretting corrosion [20,51]. Fretting of the Ti6Al4V and the Orthinox SS alloys could contribute to the statistically significant raise in Al, Ti, and Ni observed in the revision groups (Table 4) [52,53]. Highest concentrations of Co and Cr were detected in the AL (+) group. One patient in this group had a MoM implant but no markedly increased in Co or Cr concentrations were detected in either periimplant tissue or serum from this specific patient. Interestingly, relative low concentrations of Co were found in tissue and blood samples compared to Cr concentrations. This observation has previously been explained by a faster elimination of Co from both the tissue and blood than that of Cr [54]. No upper limits are currently employed to describe critical metal release from implants, but an upper limit of 7 ppb for Co and Cr in blood is often used as an action level for MoM implants [55]. Serum concentrations of this magnitude were not detected in this study. In general, our results confirm previous metal concentrations reported in serum and periimplant tissue from patients with poorly functioning implants [17]. A correlation between the metal content in periimplant tissue but not that of serum has recently been made to a lymphocyte dominated response [56]. This emphasizes the importance of the periimplant environment, in which we found highly raised metal concentrations. 

In this study implants with different fixation strategies was used i.e. cemented implants and different surface treatments for optimizing stability and osseointegration. Metal release and implant performance is highly dependent on the micro/nano topography of the implant surface [57,58]. Cemented implants have been proved to increased initial stability and minimize micro-motions of cemented parts leading to long survival rates [59]. The downside of this approach is the possible formation of a crevice between the cement and implant, which can provide a highly corrosive environment and lead to accelerated corrosion and subsequently implant failure by AL [60,61]. All uncemented implants in this study had some form of increased roughness applied to their surfaces for optimal osseointegration (Table 1). One of the costs of increasing the surface roughness on implant is an increased functional surface area, which in turn will increase metal release. Especially titanium release has recently become a subject of concern and not only in implants used for THRs [62,63,64,65]. Another debated strategy of improving osseointegration is the use of hydroxyapatite (HA) coatings, simulating the bone chemistry and structure. However, recent studies suggest that the long-term effects are not improved compared to other porous coatings or rough sandblasted surfaces [66,67].

Patch testing showed a diverse profile of test reactions across all groups making results difficult to interpret (Table 2). Metals salts are well-known skin irritants and skin reactions may therefore, in reality, be an irritant rather than an allergic reaction. On the other hand, a positive reaction can only occur if the metal reaches the viable layers of the epidermis, and this might be a challenge for some metals [68]. One patient in the AL (+) group had a positive reaction to Cr, which is higher than expected considering that less than 1% of the general population are allergic to Cr [69]. Surprisingly, we found positive reactions to Ti (IV) in the control group, which had not been exposed to Ti containing implants. Although Ti allergy is considered very limited in THRs, in vitro studies of Ti particles suggest that these can initiate innate and adaptive Th2 cell response [68,70]. Within the field of odontology there is a growing concern of the innate immune response associated with Ti, which is believed to cause osteolysis through macrophage secretion of IL1β, IL6, and TNFα [6,71]. A relative high number of skin reactions to V were observed, although most of these were scored as doubtful, true allergy cannot be ruled out. While larger cohort studies have found an increased prevalence of metal allergy in THR patients our study was not powered to examine a possible association [21,72]. Nonetheless, our findings indicate that metal allergy, as tested by patch test, is not likely to be a key driver of AL in most patients.

## 5. Conclusions

Aseptic loosening of implants is a complex tissue response influenced by various factors. Metal release from implants may generate DTH response capable of accelerating aseptic loosening of implants. In this study, we report a distinct cytokine profile in periimplant tissues between patients with implant failure due to AL, compared to mechanical causes, with statistically significant increased levels of IL-1β, IL-2, IL-4, IL-6, IL-8, IL-10, GM-CSF, IFN-γ and TNF-α. In addition, raised metal concentrations were found in blood and periimplant tissue from patients with failed THRs. Despite these observations, we failed to detect any correlation between the prevalence of metal allergy and failed THRs or AL. This work contributes to a better understanding of the immunologic nature of aseptic loosening and suggests that the immunological events involved in AL are of both innate and adaptive character.

## Figures and Tables

**Table 1:** Implant overview. Implant types and materials used for femoral, head, liner and acetabular components are given for patients in the revision groups. In addition to the implant bulk material, model names and surface finish is also listed. cpTi relates to commercially pure titanium and PS to plasma sprayed coatings. FeCrNiMn is also referred to as Orthinox stainless steel.

Patient #	Type	Femoral	Head	Liner	Acetabular
1 AL (+)	MoP	Ti-6Al-4V, ZMR®, uncemented, porous coating	CoCrMo	PE	Ti-6Al-4V, Trilogy®, uncommented, cpTi fiber mesh.
2 AL (+)	MoP	FeCrNiMn, Exeter®, cemented, polished.	CoCrMo	PE	cpTi, Duraloc®, uncemented, porous coating.
3 AL (+)	MoP	CoCrMo, Lubinus®, cemented polished.	CoCrMo	PE	PE, Lubinus®, cemented, all-polycup
4 AL (+)	MoP	FeCrNiM, Exeter®, cemented, polished.	CoCrMo	PE	Ti-6Al-4V, Mallory Head, uncemented, PS.
5 AL (+)	MoP	Ti-6Al-4V, Bi-metric®, uncemented, grit blasted.	CoCrMo	PE	Ti-6Al-4V, Mallory® Head, uncemented, PS.
6 AL (+)	MoM	Ti-6Al-4V, Bi-metric®, uncemented, grit blasted.	CoCrMo	CoCrMo	CoCrMo, ReCap®, uncemented, cpTI PS.
1 AL (−)	MoP	Ti-6Al-7Nb, CLS spotorno®, uncemented, grit blasted.	CoCrMo	PE	Ti-6Al-4V, Trilogy®, uncemented, cpTi fiber mesh.
2 AL (−)	MoP	Ti-6Al-7Nb, CLS spotorno®, uncemented, grit blasted.	CoCrMo	PE	Ti-6Al-4V, Trilogy®, uncemented, cpTi fiber mesh.
3 AL (−)	CoP	Ti-6Al-4V, Biocontact®, uncemented, grit blasted.	Ceramic	PE	Ti-6Al-4V, Plasmacup®, uncemented, plasmapore PS.
4 AL (−)	MoP	FeCrNiMn, Exeter®, cemented, polished.	CoCrMo	PE	cpTi, Pinnacle®, uncemented porocoat, porous coating.
5 AL (−)	MoP	FeCrNiMn, Exeter®, cemented, polished	CoCrMo	PE	Ti-6Al-4V, Trilogy®, uncemented, cpTi fiber mesh.
6 AL (−)	MoP	FeCrNiMn, Exeter®, cemented, polished	CoCrMo	PE	cpTi, Pinnacle®, uncemented porocoat, porous coating.

**Table 2:** Skin reactions. Positive (+) and doubtful (+?) skin reactions to different metals and methyl methacrylate. Patch test reactions were scored using the International Contact Dermatitis Research Group’s (ICDRG) criteria [30]. Only definite +1, +2 and +3 reactions were regarded as positive. No reactions were categorized as +2 and +3 reactions in this study and only compounds with either positive (+1) or doubtful (+?) reactions are listed in the table. Prevalence of positive reactions was tested against the control group using Fisher’s exact test with two tailed *p* values. No statistical significant differences were found.

	AL (+) (*n* = 6)	AL (−) (*n* = 6)	Control (*n* = 8)
	Reactions		
Metal compound (concentration)	+ (+?)	+ (+?)	+ (+?)
Al(III), AlCl_3_ (0.72%)	0 (0)	0 (0)	0 (1)
Ti(IV), TiC_4_O_8_ (0.32%)	0 (0)	1 (0)	2 (0)
Ti(II), C_4_K_2_O_9_Ti (2.4%)	0 (0)	0 (0)	0 (0)
V(III), VCl_3_ (0.24%)	1 (2)	0 (3)	0 (3)
V(III), VCl_3_ (0.12%)	1 (0)	0 (1)	0 (3)
V(III), VCl_3_ (0.013%)	0 (0)	0 (1)	0 (0)
V(III), VCl_3_ (0.04%)	0 (0)	0 (1)	0 (0)
V(IV), VOSO_4_ (0.36%)	0 (1)	0 (1)	0 (2)
V(IV), VOSO_4_ (0.18%)	0 (1)	0 (1)	0 (0)
Cr(VI), K_2_Cr_2_O_7_ (0.054%)	1 (0)	0 (0)	0 (0)
Mn(II), MnCl_2_ (0.24%)	0 (1)	1 (2)	0 (2)
Ni(II), NiSO_4_ (5.0%)	0 (0)	0 (0)	1 (1)
Methyl Methacrylate, C_5_H_8_O_2_ (2%)	0 (0)	0 (0)	0 (1)
Total reactions	3 (4)	2 (8)	3 (12)

**Table 3:** Elemental analysis. Metal concentrations (ppb) measured by ICP-MS in periimplant tissue and blood serum. Titanium, chromium and cobalt were measured in blood serum. Values are shown as group medians with interquartile range below. Statistics are based on medians using the Wilcoxon-Mann-Whitney test with a significance level of 0.05. * Indicate significantly increased values compared to the control group. Elemental analysis for Al, V and Ni was only carried out on tissue samples and are therefore indicated as not available (N/A) for serum samples.

Metal	AL (+) *n* = 6		AL (−) *n* = 6		Control *n* = 10	
	Tissue	Serum	Tissue	Serum	Tissue	Serum
**Al**	7186 * (1905–29,019)	N/A	3407 * (845–26,709)	N/A	1258 (352–2615)	N/A
**Ti**	1610 * (891–13,328)	0.65 (0.60–2.95)	12978 * (588–47,078)	1.45 (0.60–3.98)	716.5 (504–1152)	0.60 (0.60–1.00)
**V**	210 (128–920)	N/A	381 (151–573)	N/A	160 (133–209)	N/A
**Cr**	3648 (358–21,075)	0.98 * (0.26–3.4)	499 (151–6235)	0.26 (0.26–0.26)	484 (184–1868)	0.26 (0.26–0.26)
**Co**	210 (128–2724)	0.30 (0.30–1.93)	167 (118–2549)	0.30 (0.30–0.74)	160 (133–209)	0.30 (0.30–0.30)
**Ni**	772 * (355–2027)	N/A	328 (151–1589)	N/A	212 (162–326)	N/A

**Table 4:** Alloy composition. Elemental composition of the different implant alloys found patient groups, based on the ASTM international standard.

Implant Alloy	CoCrMo ASTM- (F75)	Orthinox SS ASTM- (F1586)	cpTi ASTM- (F67)	Ti6Al7Nb ASTM- (F1295)	Ti6Al4V ASTM-(F136)
Element	Composition, wt.%				
Aluminum (Al)	0.10	-	0.03	5.50–6.50	5.5–6.50
Carbon (C)	0.35	0.08	0.08	0.08	0.08
Chromium (Cr)	27–30	19.5–22	-	-	-
Cobalt (Co)	Balance	-	-	-	-
Copper (Cu)	-	0.25	0.10	-	-
Iron (Fe)	0.75	Balance	0.50	0.25	0.25
Manganese (Mn)	1	2–4.25	-	-	-
Molybdenum (Mo)	5–7	2–3	-	-	-
Nickel (Ni)	0.50	9.0–11.0	-	-	-
Niobium (Nb)	-	0.25–0.8	0.015	6.50–7.50	-
Nitrogen (N)	0.25	0.25–0.5	0.15	0.05	0.05
Oxygen (O)	-	-	0.40	0.20	0.13
Tantalum (Ta)	-	-	Balance	0.50	-
Titanium (Ti)	0.10	-	-	Balance	Balance
Tungsten (W)	0.20	-	-	-	-
Vanadium (V)	-	-	-	-	3.5–4.5
